# Synthesis of Hollow Calcium Carbonate Microspheres by a Template Method for DOX Loading and Release with Carbon Dots Photosensitivity

**DOI:** 10.3390/ma15248768

**Published:** 2022-12-08

**Authors:** Fuwang Mo, Qiujuan Chen, Xiaohui Zhang

**Affiliations:** 1 Guangxi Key Laboratory of Calcium Carbonate Resources Comprehensive Utilization, Hezhou 542899, China; 2 College of Materials and Chemical Engineering, Hezhou University, Hezhou 542899, China

**Keywords:** hollow calcium carbonate, doxorubicin hydrochloride, carbon quantum dots, drug load, sustained release

## Abstract

Calcium carbonate, as the main inorganic component of human bones and teeth, has good biocompatibility and bioactivity and finds increasing applications in the field of bone drug carriers. In this study, hollow calcium carbonate microspheres were synthesized by a water hydrothermal method using folic acid as a template. Before drug loading, the prepared calcium carbonate microspheres were subjected to aminidation, carboxylation, and vinylenimine modification. The hollow calcium carbonate microspheres loaded with doxorubicin hydrochloride (DOX) were further incorporated with light-emitting carbon quantum dots(CQDs) and hyaluronic acid (HA). The result showed that the drug loading capacity in the as-prepared calcium carbonate was 179.064 mg/g. In the simulated solutions of cellular metabolism containing various concentrations of reduced glutathione(GSH), the sustained release of DOX was confirmed qualitatively by the luminescence of the CQDs. The DOX release rate was measured quantitively by UV absorption spectra. The highest release rate reached 85.99% in a simulated solution of 0.005 mol/L GSH solution, and the release rate could vary intelligently with the concentration.

## 1. Introduction

Effective treatment of malignant tumors has always been the research target of pharmaceutical researchers. However, it remains challenging to achieve controlled release and deep delivery of drugs in tumor tissues. Previous studies have usually used tumor environment specificity, such as acid [1,2], high concentrations of glutathione [3], or highly expressed enzymes [4,5], to increase drug release. Since these initiators are consumed or saturated in the process, drug release tends to flatten out or fail over an extended time. Therefore, there is an urgent need to develop a drug delivery system that can respond to substances widely present in the tumor environment, such as water, to further improve the effective release of drugs.

Hollow calcium carbonate microspheres have high specific surface area and pore volume, good dispersion ability, and excellent biocompatibility [6,7]. They have been widely used in analytical chemistry, drug loading, and biomineralization. Li et al. [8] used tetraethylenepentamine-graphene (rGO-TEPA)/CaCO_3_ solid microspheres as intermediates to prepare composite microspheres via a microwave hydrothermal method. The doxorubicin(DOX) loading and release results revealed that the prepared carriers had mild storage−release behaviors and good pH responses. The compound drug loading system could be dispersed in a buffer solution with a pH value of 7.4, and the drug release was 9.2% in the first 4 h and reached 13.5% after 48 h. In the study of Tang [9] et al., nano-CaCO_3_ was encapsulated in a cell membrane to prepare biomimetic nanoparticles for screening bioactive molecules in T. wilfordii. This CaCO_3_ nanocomposite was demonstrated as an ideal affinity screening platform because it could screen the bioactive molecules and their target proteins to achieve a none-loss target release. To obtain stable suspensions of porous CaCO_3_ particles, various organic compounds are used as additives during the synthesis. Yang et al. [10] developed a one-pot and L-lysine (Lys)-mediated biomineralization method using a CO_2_ bubbling procedure to prepare CaCO_3_-based composites in a green, simple, and quick way. The resulting drug delivery system showed high drug loading capacity, good biocompatibility, pH sensitivity, and low toxicity. The preparation of CaCO_3_ drug carriers usually adopts the template method; that is, in the presence of micro- or nano-scale functional templates, calcium and carbonate ions react to form CaCO_3_. The shape and size of the template can significantly impact the structure and size of the resulting CaCO_3_ drug carrier.

Many researchers have investigated drug-loaded calcium carbonate carriers [11,12,13,14,15,16,17,18]. However, monitoring drug loading and release is generally achieved by some complex techniques. It is necessary to develop a convenient and fast way to detect drug loading and release properties. Carbon quantum dots (CQDs) are a type of quasi-zero-dimensional carbon material with unique photoluminescence properties due to their nanometer size. They have excellent biocompatibility and low toxicity. Especially, they can emit strong and stable fluorescence when excited by a light source. Their fluorescence intensity, lifetime, and multi-color fluorescence properties do not deteriorate after being stored for several months under normal conditions. A CQD-based prodrug nanotheranostics was synthesized by Li et al. [19] with a DOX content of 14.57 % and a mean hydrodynamic diameter of approximately 116 nm. It showed excellent pH-triggered drug release performance with a cumulative release of 52.71 % in the simulated tumor intracellular microenvironment in 4 days. CQDs and poly(acrylic acid) (PAA) were employed to modify the crystallization and assembly of CaCO_3_ by Lauth et al. [20]. The hybrid nanocarriers demonstrated excellent colloidal stability in cell medium, low cytotoxicity, high loading efficiency (approximately 30% for Rhodamine B), and pH-controlled release. Despite the unique luminous properties of CQDs [21,22,23,24,25], there are limited reports combining CQDs with inorganic solids for drug delivery.

In this study, we used folic acid (FA) as a template and CaCl_2_ and Na_2_CO_3_ as raw materials to synthesize hollow CaCO_3_ microspheres by a hydrothermal method. After surface modification, the CaCO_3_ microspheres were loaded with DOX to investigate the drug-loading capacity. The drug release properties were explored in cell simulants containing different concentrations of reduced glutathione (GSH).

## 2. Results

**Figure 1:**
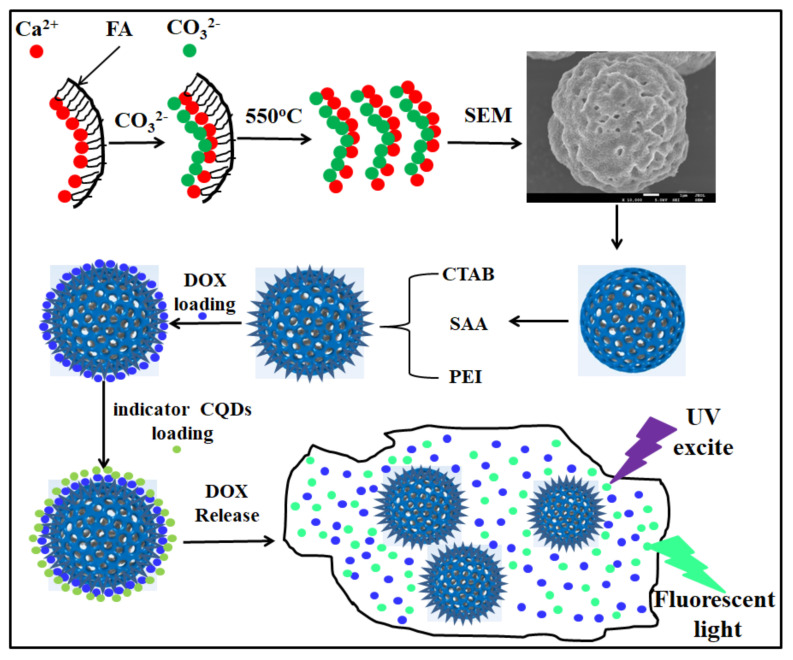
Schematic diagram of the preparation of FA-CaCO_3_/PEI@DOX hollow calcium carbonate microspheres.

As shown in Figure 1, FA-CaCO_3_ hollow microspheres were prepared via a hydrothermal method and further soaked in hexadecyltrimethyl ammonium bromide (CTAB), succinic anhydride (SAA), and polyethyleneimine (PEI) for aminidation, carboxylation, and vinylenimine modification, respectively, before drug loading.

**Figure 2:**
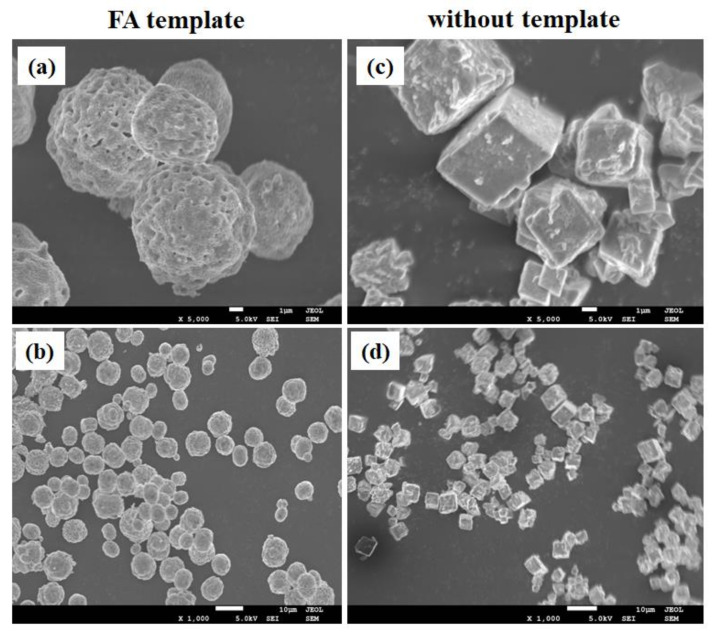
SEM images of (**a**,**b**) FA-CaCO_3_ and (**c**,**d**) CaCO_3_ prepared without a template.

Morphologies of the CaCO_3_ microspheres prepared with and without FA template were observed using scanning electron microscopy (SEM). As shown in Figure 2a,b, porous microsphere structures were obtained in the presence of FA, wherein the diameter of the microsphere was between 7 and 8 μm, and the pore size was between 0.3 and 0.5 μm. In contrast, the CaCO_3_ microspheres prepared without an FA template showed regular cube structures, and no holes were observed on the surface (Figure 2c,d).

**Figure 3:**
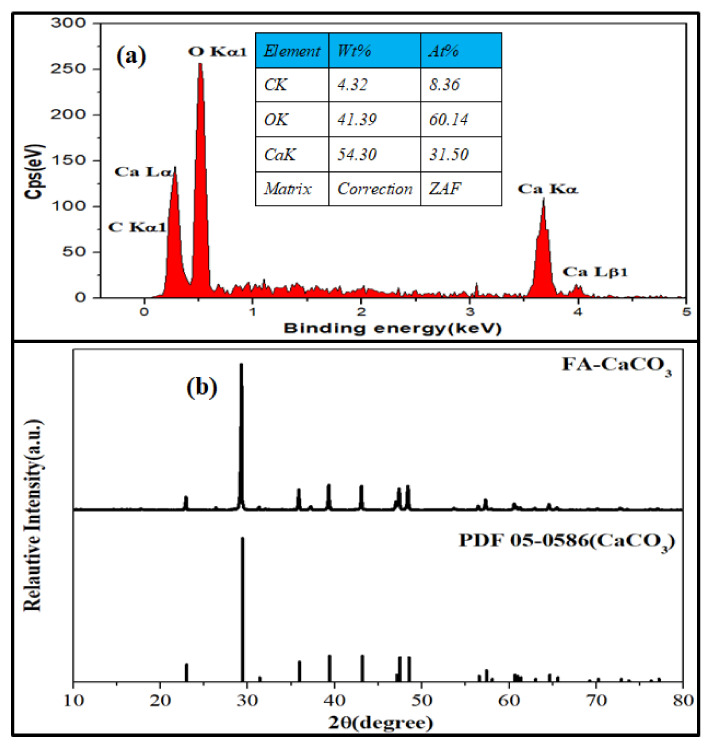
(**a**) EDS and (**b**) XRD pattern of FA-CaCO_3_.

The composition and crystal structure of the FA-CaCO_3_ were investigated using energy-dispersive X-ray spectroscopy (EDS) and X-ray diffraction (XRD), respectively. As shown in Figure 3a, the atomic percentages of carbon, oxygen, and calcium in the selected area were 8.36, 60.14, and 31.50%, respectively. The XRD pattern (Figure 3b) suggested that the crystal structure of the FA-CaCO_3_ agreed with that of Rhombohedral CaCO_3_.

**Figure 4:**
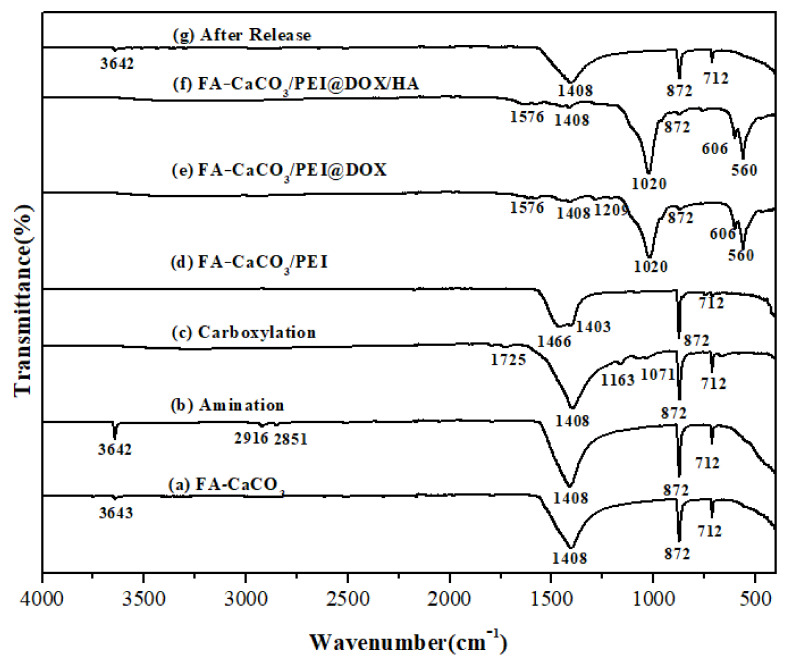
FT-IR spectra of the (**a**)FA-CaCO_3_, (**b**) Aminated FA-CaCO_3_, (**c**) Carboxylated FA-CaCO_3_, (**d**) FA-CaCO_3_/PEI, (**e**) FA-CaCO_3_/PEI@DOX, (**f**) FA-CaCO_3_/PEI@DOX/HA, and (**g**) CaCO_3_ microspheres after drug release.

The Fourier-transform infrared spectroscopy (FT-IR) spectra of the composite microspheres are shown in Figure 4. The absorption bands at 1408, 873, and 712 cm^−1^ in FA-CaCO_3_ before DOX loading (Figure 4a) and FA-CaCO_3_ after DOX release (Figure 4g) were related to CaCO_3_ calcite [26,27]. Meanwhile, in Figure 4b, the peak at 3643 cm^−1^ was strengthened compared with Figure 4a because of the N-H groups, and the new absorption peaks at 2916 cm^−1^ and 2851 cm^−1^ were ascribed to the –CH_2_ groups in the CTAB. The peaks at 1725, 1163, and 1071 cm^−1^ in Figure 4c corresponded to the -CHO, -C-O-, and C-OH groups, respectively. For FA-CaCO_3_ encapsulated by PEI, the -CH_2_- groups in the long PEI chain showed a peak at 1466 cm^−1^ (Figure 4d). When the drug (DOX) was loaded, a strong absorption emerged at 1020 cm^−1^, corresponding to the primary alcohol structure in the DOX molecule (Figure 4e). After loading CQDs, new absorption peaks were detected at 606 and 560 cm^−1^ due to the benzene structure (Figure 4f) [28,29,30]. These results indicated that both drug (DOX) and indicator (CQDs) were successfully loaded on the surface of FA-CaCO_3_/PEI.

**Figure 5:**
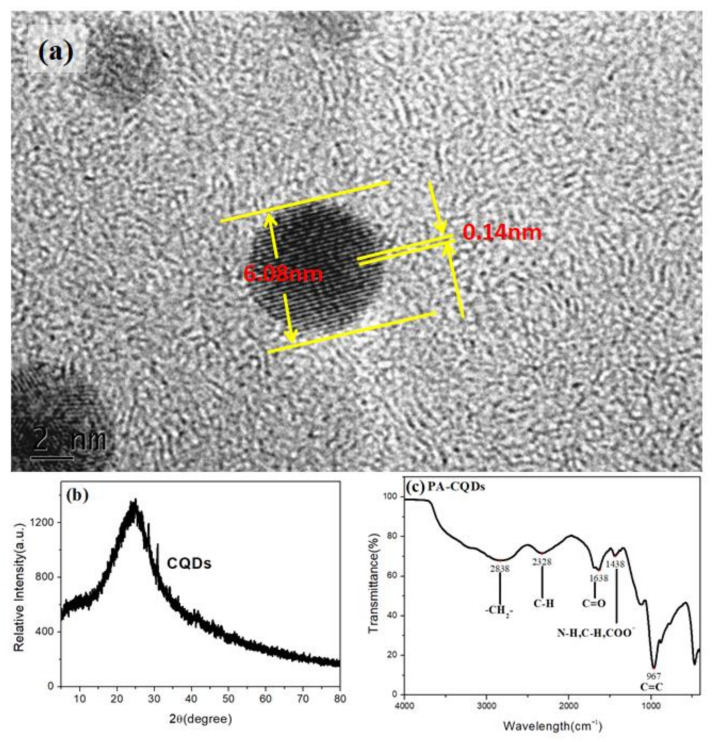
(**a**) TEM, (**b**) crystal structure, and (**c**) FTIR spectrum of CQDs.

The morphologies, structures, and luminescence properties of the CQDs were further studied using Transmission Electron Microscope(TEM) and FT-IR spectra (Figure 5). As shown in Figure 5a, the TEM images on the particles showed a lattice stripe distribution, suggesting a regular arrangement of carbon materials (CQDs). At the same time, the lattice stripes displayed an interlayer distribution with a spacing of approximately 0.14 nm, which matched the (0 2 0) crystal plane of graphene. As shown in the XRD pattern (Figure 5b), the diffraction peak at about 24° was associated with the (0 2 0) crystal plane, which was consistent with the TEM image [24,31,32]. In the FT-IR spectra (Figure 5c), the peak at 967 cm^−1^ was ascribed to the out-of-plane bending vibration of C=C. The peaks at 1038 cm^−1^, 1638 cm^−1^, 2328 cm^−1^, and 2838 cm^−1^ were caused by the stretching vibrations of C-O, C=O, C≡N, and –CH_2_-, respectively. The results implied that the prepared CQDs surface was highly hydrophilic and enriched with abundant hydrophilic groups.

**Figure 6:**
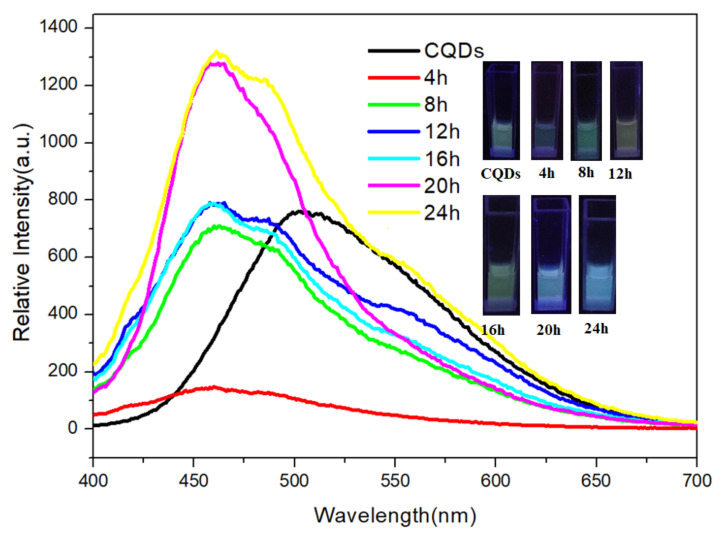
Emission spectra (λ_ex_ = 365 nm) of CQDs at different time.

In this study, a cell simulation solution with a reduced glutathione (GSH) concentration of 0.0001 mol/L was prepared for the fluorescence test. The fluorescence emission spectra of CQDs at different release times are shown in Figure 6. The luminescence peak of CQDs in the GSH solution showed a blue shift as the release time, and the intensity decreased first then increased. It was because the slowly released DOX coated the CQD surface, repaired the surface defect, and made the CQDs particles smaller. The inset in Figure 6 shows the color change from 0 to 24 h, which could be a qualitative indicator of the DOX release characteristics.

**Figure 7:**
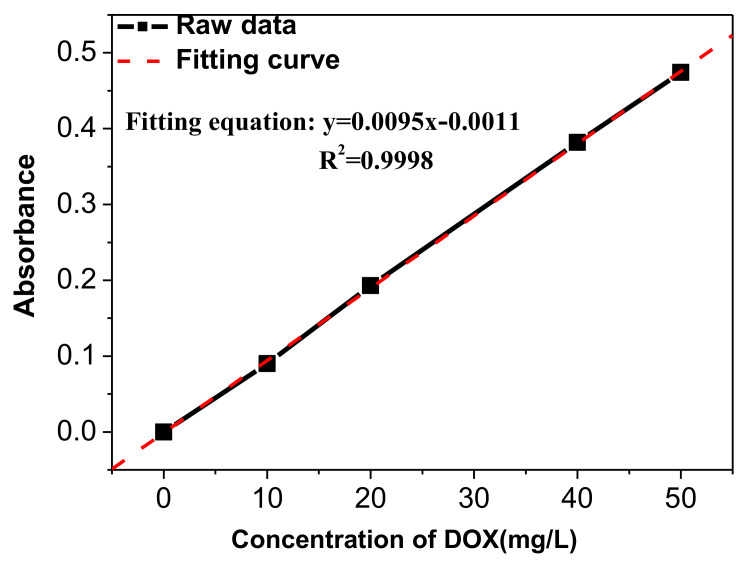
UV absorption standard curve of DOX.

During the loading experiment, the FA-CaCO_3_/PEI microspheres were placed in a cell microenvironment release solution, i.e., an oxidized glutathione (GSSG) solution, for solid surface modification in the cell environment. Then, DOX was loaded to the microspheres in the buffer solution to study the loading behavior. Figure 7 shows the Ultraviolet and Visible (UV) absorption standard curve of DOX.

The UV absorption standard curve of DOX had a linear relationship of

*y* = 0.0095 x − 0.0011 (1)

where *y* is the average absorbance, and x is the concentration of DOX solution (mg/L). After placing 0.0707 g of FA-CaCO_3_/PEI in 1 mg/mL of DOX solution for 24 h, the absorbance of the supernatant was 1.481, which was converted to a concentration of 156.01 mg/L according to Formula (1). The factor of drug loading (*F*) was calculated by the following equation:

(2) $$F=\frac{m_{T}-m_{S}}{m_{C}}\times 100\%=\frac{c_{o}\times V_{o}-c_{S}\times V_{o}}{m_{C}}\times 100\%$$

where *m_T_* is the total mass of DOX, *m_S_* is the mass of DOX dispread in the supernatant, m_C_ is the mass of FA-CaCO_3_/PEI (0.0707 g), *c_o_* is the original concentration of DOX solution (1 mg/mL), *V_o_* is the volume of the DOX solution (15 mL), *c_s_* is the concentration of DOX in the supernatant (calculated as 156.01 mg/L based on Equation (1)). Using Equation (2), the *F* value was calculated as 179.064 mg/g, i.e., the adsorption capacity was 15.19 wt.%.

The FA-CaCO_3_/PEI microspheres were further loaded with hyaluronic acid (HA). Then, FA-CaCO_3_/PEI@DOX/HA (10 mg) was dispersed in 10 mL of PBS buffer solution (pH = 7.4) and poured into a dialysis bag (molecular weight cut-off = 4000). The bag was placed in GSH buffers of different concentrations (pH = 7.4) and tested for DOX release at 4 h intervals. The results are listed in Table 1. When the tumor microenvironment (GSH) concentration increased, more DOX was released. The highest release rate reached 85.99%, more than eight times higher than the GSH-free solution. In all the tested tumor cell stimulation fluids, DOX release could maintain steady and slow within 24 h, and DOX release increased with the concentration of the GSH. It indicated that the drug release could be modulated by the change of tumor cell environment, showing the potential of intelligent drug release.

## 3. Materials and Methods

Materials. Na_2_CO_3_ (99%), CaCl_2_ (99%), and o-phenylenediamine(OPD, 99%) were purchased from Xilong Chemical Co., Ltd (Shantou, China). Toluene was obtained from Sinopharm Group Chemical Reagent Co., Ltd (Shanghai, China). Folic acid (FA, 99%), Hexadecyltrimethyl ammonium Bromide (CTAB, 99%), N,N-Dimethylformamide (DMF, 99.5%), Succinic anhydride(SAA,98%), N-Hydroxy succinimide (NHS, 98%), Triethanolamine (TEA, 98%), 1-(3-Dimethylaminopropyl)-3-ethylcarbodiimide hydrochloride (EDC, 98.5%), Polyethyleneimine (PEI, 99.7%), oxidized glutathione (GSSG, 98%), reduced glutathione (GSH, 98%), and hyaluronic acid (HA, 97%) were purchased from Shanghai Macklin Biochemical Co., Ltd (Shanghai, China). The buffer solutions (PBS, pH = 7.4) were obtained from Jiangbiao Testing Technology Co., Ltd., Nanjing, China.

Preparation of the composite calcium carbonate. CaCl_2_ (0.111 g) and folic acid (0.1 g) were dissolved in 25 mL of deionized water to form solution A. Sodium carbonate (0.106 g) and folic acid (0.1 g) were dissolved in 25 mL of deionized water to form solution B. Then, solution B was slowly added to solution A within an hour and aged for another hour. The mixed solution was placed in a reactor kettle at 200 °C for 24 h. The product was cooled, filtered, washed with alcohol 3 times, and dried at 80 °C to obtain the FA-CaCO_3_ precursor. The precursor was calcined at 550 °C for 2 h to receive FA-CaCO_3._ The as-prepared FA-CaCO_3_ was dispersed in 20 mL of toluene for 1 h, then 2 mL of CTAB was added. The mixture was stirred for 0.5 h, then filtered, washed with alcohol 3 times and dried at 60 °C to obtain amininated calcium carbonate microspheres. The above aminidated microspheres were dispersed in 35 mL of DMF with the assistance of ultrasound for 5 min, followed by adding 5 mL of SAA and 0.9 mL of TEA solutions. The mixture was stirred for 24 h, centrifugated, washed with alcohol, and dried at 60 °C to obtain carboxylated microspheres. The carboxylated microspheres were dispersed for 5 min in 20 mL of DMF, then 32 mL of EDC and 20 mL of NHS were added and stirred for 0.5 h. Next, 3 mL of PEI solution (0.1 mg/mL) was added and stirred for 5 h. The product was washed, dried at 60 °C and sonicated in a GSSG solution for 5 min. The GSSG solution was stirred for 12 h, filtered, and finally dried at 30 °C to obtain the FA-CaCO_3_/PEI for drug loading.

Preparation of carbon quantum dots(CQDs). OPD (0.1 g) and FA (0.1 g) were dispersed in 10 mL of ethanol and stirred for 30 min. The mixture was poured into a reaction kettle and reacted at 200 °C for 24 h. After natural cooling, the product was put into a dialysis bag (molecular weight cut-off = 4000) and dialyzed in deionized water for 24 h. The product was freeze-dried to obtain solid CQDs.

DOX loading Experiments. FA-CaCO_3_/PEI (0.0707 g) was dispersed in 15 mL of DOX solution (1 mg/mL) in PBS (pH = 7.4 and 5.8) at 37 °C in a water bath shaker (100 r/min) for 24 h. The product was centrifuged, washed, and dried to obtain the CaCO_3_ microsphere carrier (FA-CaCO_3_/PEI@DOX). The supernatant was collected and tested using a UV-visible spectrophotometer at an absorbance of 480 nm. The loaded drug was calculated according to the DOX standard solution curve. The FA-CaCO_3_/PEI@DOX microspheres were dispersed in 5 mL PBS solutions (pH = 7.4) containing 30 mg of EDC and 40 mg of NHS and stirred for 30 min. Then, 1 mg of HA and 15 mg of CQDs were added to the solutions, centrifuged, and dried at 30 °C to obtain a porous CaCO_3_ drug vehicle treated by HA blocking (FA-CaCO_3_/PEI@DOX/HA). The supernatant was collected and tested using a fluorescence spectrophotometer.

DOX Release Experiments. FA-CaCO_3_/PEI@DOX/HA (10 mg) was dispersed in 10 mL of PBS (pH = 7.4), and the solution was placed in a dialysis bag (molecular weight cut-off = 4000). The solution was placed in 10 mL of GSH buffer solutions of different concentrations (pH = 7.4) in a water bath shaker (100 r/min) at 36.8 °C. The absorbance was measured by a UV-visible spectrophotometer and a fluorescence spectrophotometer at the same time. Parallel experiments were performed three times for each group.

Characterization. The morphologies of the microspheres were observed with a 7610F SEM. The crystal structures were analyzed on a Rigaku U4 powder XRD in the 2θ range from 10 to 80° with a step size of 0.02°. TEM images were obtained on a Tecnai G2 F20 TEM system. The DOX concentration was determined using UV-Vis and fluorescence spectrophotometers.

## 4. Conclusions

In summary, spherical CaCO_3_ microspheres with hollow structures were successfully prepared via a conventional hydrothermal method. SEM results showed that CaCO_3_ with a regular cube structure could be obtained without using FA as a template during the synthesis procedure, while CaCO_3_ microspheres with a hollow structure formed in the presence of FA. FT-IR spectra indicated that CTAB, SAA, and PEI could successfully modify the as-prepared hollow calcium carbonate micropheres wihout influencing their inherent characteristics and both the drug (DOX) and hydrophilic indicator (CQDs) could be loaded on the surface of FA-CaCO_3_/PEI. The luminescence variations of the CQDs could be served as an indicator of the release of DOX, and the release time can be as long as 24 h with a steady and slow speed. The calculated sorption capacity of DOX could reach 15.19 wt.%, and its highest release rate was 85.99% in a simulated solution of 0.005 mol/L GSH solution, which was related to the concentration of GSH. All above results suggested that the FA-CaCO_3_/PEI@DOX/HA particles have great potential in delivering toxic anticancer drugs.

## Figures and Tables

**Table 1:** Summary of drug release data.

Concentration of GSH (mol/L)	Time (h)	Abs	Concentration of DOX (mg/L)	F (%)
0	4	0.034	3.695	9.73%
	8	0.064	6.853	18.05%
	12	0.064	6.853	18.05%
	16	0.074	7.905	20.82%
	20	0.061	6.537	17.22%
	24	0.067	7.168	18.88%
0.0001	4	0.071	7.589	20.38%
	8	0.075	8.011	21.51%
	12	0.096	10.221	27.44%
	16	0.099	10.537	28.29%
	20	0.084	8.958	24.05%
	24	0.067	7.168	19.25%
0.001	4	0.128	13.589	35.80%
	8	0.144	15.274	40.24%
	12	0.193	20.432	53.83%
	16	0.171	18.116	47.72%
	20	0.189	20.011	52.72%
	24	0.169	17.905	47.17%
0.005	4	0.399	42.116	73.50%
	8	0.413	43.589	76.07%
	12	0.4	42.221	73.68%
	16	0.439	46.326	80.85%
	20	0.467	49.274	85.99%
	24	0.444	46.853	81.77%

## Data Availability

Data available on request from the authors.

## References

[1] Ding H., Tan P., Fu S., Tian X., Zhang H., Ma X., Gu Z., Luo K. (2022). Preparation and application of pH-responsive drug delivery systems. J. Control Release.

[2] Yang X., Wu W., Li J., Hu Z., Wang N., Yu X. (2020). A facile strategy to construct fluorescent pH-sensitive drug delivery vehicle. Polymer.

[3] Yu K., Hai X., Yue S., Song W., Bi S. (2021). Glutathione-activated DNA-Au nanomachine as targeted drug delivery platform for imaging-guided combinational cancer therapy. Chem. Eng. J..

[4] Liu F., Wang D., Zhang M., Ma L., Yu C., Wei H. (2022). Synthesis of enzyme-responsive theranostic amphiphilic conjugated bottlebrush copolymers for enhanced anticancer drug delivery. Acta Biomater..

[5] Fouladi F., Steffen K.J., Mallik S. (2017). Enzyme-Responsive Liposomes for the Delivery of Anticancer Drugs. Bioconjug. Chem..

[6] Zheng A., Zhu S., Zhou J., Wang H. (2022). Dopamine- and citrate-mediated, rapid synthesis of hollow calcium carbonate nanoparticles: Their formation, metastability and transformation. Colloids Surfaces A.

[7] Zheng T., Yi H., Zhang S., Wang C. (2020). Preparation and formation mechanism of calcium carbonate hollow microspheres. J. Cryst. Growth.

[8] Li J., Liu M., Qiu Y., Gan Y., Jiang H., Liu B., Wei H., Ma N. (2021). Urchin-like hydroxyapatite/graphene hollow microspheres as pH-responsive bone drug carriers. Langmuir.

[9] Tang Y., Qian W., Zhang B., Liu W., Sun X., Ji W., Ma L., Zhu D. (2021). None-loss target release of biomimetic CaCO_3_ nanocomposites for screening bioactive components and target proteins. ACS Appl. Bio Mater..

[10] Yang T., Ao Y., Feng J., Wang C., Zhang J. (2021). Biomineralization inspired synthesis of CaCO_3_-based DDS for pH-responsive release of anticancer drug. Mater. Today Commun..

[11] Liu Y., Ma X., Zhu Y., Lv X., Wang P., Feng L. (2022). pH-responsive nanomedicine co-encapsulated with erlotinib and chlorin e6 can enable effective treatment of triple negative breast cancer via reprogramming tumor vasculature. Chem. Eng. J..

[12] Tan H., Liu Y., Hou N., Cui S., Liu B., Fan S., Yu G., Han C., Zheng D., Li W. (2022). Tumor microenvironment pH-responsive pentagonal gold prism-based nanoplatform for multimodal imaging and combined therapy of castration-resistant prostate cancer. Acta Biomater..

[13] Manabe K., Oniszczuk J., Michely L., Belbekhouche S. (2020). pH- and redox-responsive hybrid porous CaCO_3_ microparticles based on cyclodextrin for loading three probes all at once. Colloids Surf. A Physicochem. Eng. Asp..

[14] Liu Y., Li Y., Xue G., Cao W., Zhang Z., Wang C., Li X. (2021). Shape switching of CaCO_3_-templated nanorods into stiffness-adjustable nanocapsules to promote efficient drug delivery. Acta Biomater..

[15] Eurov D.A., Kurdyukov D.A., Boitsov V.M., Kirilenko D.A., Shmakov S.V., Shvidchenko A.V., Smirnov A.N., Tomkovich M.V., Yagovkina M.A., Golubev V.G. (2022). Biocompatible acid-degradable micro-mesoporous CaCO_3_:Si:Fe nanoparticles potential for drug delivery. Micropor. Mesopor. Mater..

[16] Dou J., Zhao F., Fan W., Chen Z., Guo X. (2020). Preparation of nonspherical vaterite CaCO_3_ particles by flash nanoprecipitation technique for targeted and extended drug delivery. J. Drug Deliv. Sci. Technol..

[17] Wei Y., Sun R., Su H., Xu H., Zhang L., Huang D., Liang Z., Hu Y., Zhao L., Lian X. (2021). Synthesis and characterization of porous CaCO_3_ microspheres templated by yeast cells and the application as pH value-sensitive anticancer drug carrier. Colloids Surf. B Biointerfaces.

[18] Feng Z., Yang T., Dong S., Wu T., Jin W., Wu Z., Wang B., Liang T., Cao L., Yu L. (2022). Industrially synthesized biosafe vaterite hollow CaCO_3_ for controllable delivery of anticancer drugs. Mater. Today Chem..

[19] Li G., Pei M., Liu P. (2020). DOX-conjugated CQD-based nanosponges for tumor intracellular pH-triggered DOX release and imaging. Colloids Surf. A Physicochem. Eng. Asp..

[20] Lauth V., Loretz B., Lehr C.-M., Maas M., Rezwan K. (2016). Self-assembly and shape control of hybrid nanocarriers based on calcium carbonate and carbon nanodots. Chem. Mater..

[21] Najaflu M., Shahgolzari M., Bani F., Khosroushahi A.Y. (2022). Green synthesis of near-infrared copper-doped carbon dots from alcea for cancer photothermal therapy. ACS Omega.

[22] Gautam B., Huang M.-R., Ali S.A., Yan A.-L., Yu H.H., Chen J.-T. (2022). Smart thermoresponsive electrospun nanofibers with on-demand release of carbon quantum dots for cellular uptake. ACS Appl. Mater. Interfaces.

[23] Wolski P. (2021). Molecular Dynamics simulations of the pH-dependent adsorption of doxorubicin on carbon quantum dots. Mol. Pharm..

[24] Su W., Guo R., Yuan F., Li Y., Li X., Zhang Y., Zhou S., Fan L. (2020). Red-emissive carbon quantum dots for nuclear drug delivery in cancer stem cells. J. Phys. Chem. Lett..

[25] Han C., Zhang X., Wang F., Yu Q., Chen F., Shen D., Yang Z., Wang T., Jiang M., Deng T. (2021). Duplex metal co-doped carbon quantum dots-based drug delivery system with intelligent adjustable size as adjuvant for synergistic cancer therapy. Carbon.

[26] Ajikumar P.K., Wong L.G., Subramanyam G., Lakshminarayanan R., Valiyaveettil S. (2005). Synthesis and characterization of monodispersed spheres of amorphous calcium carbonate and calcite spherules. Cryst. Growth Des..

[27] Mihai M., Damaceanu M.-D., Aflori M., Schwarz S. (2012). Calcium carbonate microparticles growth templated by an oxadiazole-functionalized maleic anhydride-co-N-vinyl-pyrrolidone copolymer, with enhanced pH stability and variable loading capabilities. Cryst. Growth Des..

[28] Barhoum A., Rahier H., Abou-Zaied R.E., Rehan M., Dufour T., Hill G., Dufresne A. (2014). Effect of cationic and anionic surfactants on the application of calcium carbonate nanoparticles in paper coating. ACS Appl. Mater. Interfaces.

[29] Yaseen S.A., Yiseen G.A., Li Z. (2019). Elucidation of calcite structure of calcium carbonate formation based on hydrated cement mixed with graphene oxide and reduced graphene oxide. ACS Omega.

[30] Yang T., He R., Nie G., Wang W., Zhang G., Hu Y., Wu L. (2018). Creation of hollow calcite single crystals with CQDs: Synthesis, characterization, and fast and efficient decontamination of Cd(II). Sci. Rep..

[31] Song D., Tian J., Xu W., Wen H., Wang C., Tang J., Zhang J., Guo M. (2021). Optically induced insulator-to-semiconductor transition in fluorescent carbon quantum dots measured by terahertz time-domain spectroscopy. Carbon.

[32] Wang L., Li W., Yin L., Liu Y., Guo H., Lai J., Han Y., Li G., Li M., Zhang J. (2020). Full-color fluorescent carbon quantum dots. Sci. Adv..

